# Elevational spatial compounding for enhancing image quality in echocardiography

**DOI:** 10.1177/1742271X16632283

**Published:** 2016-03-01

**Authors:** Antonios Perperidis, Norman McDicken, Tom MacGillivray, Tom Anderson

**Affiliations:** 1 Institute of Sensors, Signals and Systems, Heriot Watt University, Edinburgh, UK; 2 Medical Physics and Medical Engineering, University of Edinburgh, Edinburgh, UK; 3 Clinical Research Imaging Centre, University of Edinburgh, Edinburgh, UK; 4 Centre for Cardiovascular Science, University of Edinburgh, Edinburgh, UK

**Keywords:** Echocardiography, image enhancement, noise suppression, compounding, left-ventricle phantom

## Abstract

**Introduction:**

Echocardiography is commonly used in clinical practice for the real-time assessment of cardiac morphology and function. Nevertheless, due to the nature of the data acquisition, cardiac ultrasound images are often corrupted by a range of acoustic artefacts, including acoustic noise, speckle and shadowing. Spatial compounding techniques have long been recognised for their ability to suppress common ultrasound artefacts, enhancing the imaged cardiac structures. However, they require extended acquisition times as well as accurate spatio-temporal alignment of the compounded data. Elevational spatial compounding acquires and compounds adjacent partially decorrelated planes of the same cardiac structure.

**Methods:**

This paper employs an anthropomorphic left ventricle phantom to examine the effect of acquisition parameters, such as inter-slice angular displacement and 3D sector angular range, on the elevational spatial compounding of cardiac ultrasound data.

**Results and conclusion:**

Elevational spatial compounding can produce substantial noise and speckle suppression as well as visual enhancement of tissue structures even for small acquisition sector widths (2.5° to 6.5°). In addition, elevational spatial compounding eliminates the need for extended acquisition times as well as the need for temporal alignment of the compounded datasets. However, moderate spatial registration may still be required to reduce any tissue/chamber blurring side effects that may be introduced.

## Introduction

Echocardiography provides a valuable, real-time diagnostic tool capable of acquiring cardiac images of high spatial and temporal resolution at relatively low operational cost.^1^ However, transthoracic, cardiac ultrasound images are often incomplete (partial heart coverage) and suffer from a range of artefacts. More precisely, acoustic noise combined with speckle, the phenomenon responsible for the granular appearance of ultrasound images,^2–4^ can sometimes obscure fine anatomic detail limiting the delineation of cardiac structures. Furthermore, reverberations and shadowing, which obscure portions of the imaged structure,^1,5^ may appear momentarily or alter their position and orientation throughout a scan due to the patient’s respiratory motion. Most such artefacts are a consequence of the interaction of the ultrasound signals with anatomical structures such as bone or lungs (air).^1,5^ Such structures have a direct effect on the echogenicity of the examined patient. While advances in data acquisition technologies have substantially improved cardiac ultrasound data, a considerable portion of cardiac scans provide low-quality images of limited diagnostic value. Consequently, there is research interest in the development of effective post-processing methods that address these limitations.

Over the years, a number of approaches for enhancing cardiac ultrasound images have been suggested. Spatial compounding combines partially decorrelated images whose speckle patterns have been modified by imaging the target region of interest from different viewing angles. During spatial compounding, no potentially valuable clinical information is filtered out. Instead, tissue structures that are present in all the partially decorrelated views of the scanned structure are enhanced, while artefacts that are not present in all views are suppressed. Recent advances in data acquisition technologies, such as matrix transducers,^6–9^ enable the acquisition of real-time, non-gated, 4D cardiac ultrasound data through a single acoustic window. Numerous studies have successfully employed spatial compounding through transducer repositioning (multiple acoustic windows) for the enhancement of such 3D/4D cardiac ultrasound data.^10–16^ Other studies have attempted to utilise the repeated rhythmic contractions of the heart in order to acquire multiple 2D images of the same cardiac phase over consecutive cardiac cycles through a single acoustic window. Minor movements during a multi-cycle image acquisition alter the scan plane resulting in partially decorrelated views of the imaged cardiac structure.^17–19^ Spatially compounding such partially decorrelated frames corresponding to the same cardiac phase can therefore produce enhanced cardiac images.

The benefits of spatial compounding on cardiac ultrasound images have long been recognised,^17,19–24^ including (i) considerable speckle and noise suppression, (ii) tissue enhancement of visually weak cardiac structures and (iii) increasing the sometimes limited field of view (more complete coverage of the imaged structure). On the other hand, lengthy data acquisition is required imposing minor rules and restrictions on the data acquisition procedure. Furthermore, the main challenge for effective spatial compounding lies in the accurate and robust spatio-temporal alignment of corresponding frames/volumes acquired over different cardiac cycles and potentially different acoustic windows (in the case of compounding 3D/4D volumes). The task is amplified due to the noisy and constantly moving (and deforming) nature of cardiac ultrasound data. Inaccurate alignment may result in severe blurring of the boundaries of the imaged cardiac structure.

Elevational spatial compounding (ESC)^25^ provides an early attempt to compound 3D ultrasound data acquired by steering the imaging plane using small inclinations along the elevation plane to generate an enhanced 2D dataset. The technique was introduced and preliminarily validated on simulated data of spherical lesions and demonstrated increased contrast to noise ratio (CNR), reduction of speckle and enhancement of lesion detectability.^25^ State of the art 4D acquisition technologies (e.g., matrix transducers) can be employed to enable the real-time acquisition and compounding of the required adjacent imaging planes, minimising the need for complex spatio-temporal alignment of multiple frame/volume sequences. This paper extends the notion of ESC for use in cardiac ultrasound data. Furthermore, a controlled environment, consisting of a custom acquisition setup and an anthropomorphic left ventricle phantom, have been developed enabling the in vitro quantitative assessment of the effect of ESC on (i) cardiac tissue speckle, (ii) cardiac chamber noise, (iii) detectability between cardiac tissue and chambers and (iv) boundary blurring between cardiac tissue and chambers. The effect of acquisition parameters such as inter-slice angular displacement and 3D sector angular range has also been quantitatively assessed.

## Methods

### Scanning setup

B-mode frame sequences over adjacent slices (along the elevation plane) were acquired using a mechanically displaced 2D phased array cardiac probe. The 2D probe was attached to an RS 351-4647 unipolar geared stepper motor. Each slice was offset slightly from the previous one by a small angular displacement, θ° (pivot point at the distal end of the transducer). The collection of adjacent slices formed a thin angular 3D sector of a volumetric pyramid scan. In a clinical setup, a 4D matrix transducer can be used for the real-time, simultaneous acquisition of the adjacent slices. However, with the available acquisition setup, a manually controlled displacement of a 2D probe enabled the investigation of optimal acquisition parameters such as inter-slice angular displacement and 3D sector angular width. Such parameters have a direct effect on the signal to noise ratio (SNR) increase as well as the tissue boundary blurring introduced to the compounded data. Moreover, wide angular sector acquisition lessens the effectiveness of ESC on patients with restricted acoustic windows through the rib cage and lungs. Therefore, the objective was to introduce as great a SNR increase as possible while keeping the acquisition sector’s width to a minimum.

### Anthropomorphic left ventricle phantom

An easily reproducible and low-cost anthropomorphic left ventricle (LV) phantom was designed and developed to provide a controlled environment for the quantitative analysis and evaluation of ESC. More precisely, the phantom enabled the investigation of the relation between the angular width of the volumetric scanning sector against the SNR increase and tissue boundary blurring introduced to the scanned structure during ESC. It also enabled the examination of the relationship between inter-slice angular displacement and SNR increase.

**Figure 1.**
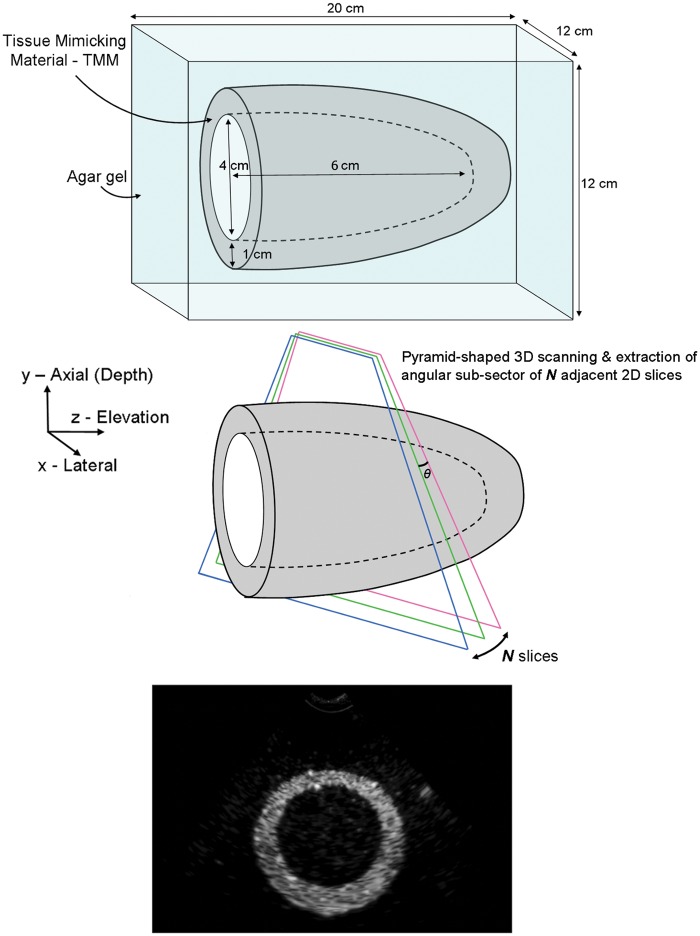
Left ventricle phantom. TMM phantom enclosed in agar gelatine filled container (for illustration purposes only – not to the correct scale). Volumetric scanning is performed by a series of adjacent 2D slices along the *x-y* plane simulating a parasternal short-axis slice of an adult human left ventricle.

A number of anthropomorphic cardiac ultrasound phantoms have been proposed in the past.^26–34^ The anthropomorphic cardiac phantom developed and employed in this study provided a simple anatomic representation of an adult human LV. More precisely, the phantom consisted of a cup-shaped (prolate-ellipsoid) tissue mimicking material (TMM) model of an adult human LV positioned along the elevation plane within a bloc of agar-based gelatin (Figure 1). The TMM^35^ provided tissue equivalence using glycerol to match the speed of sound in human tissue (1540 m/s) as well as particles such as silicon carbide (SiC) and aluminium oxide (Al_2_O_3_) to modify the ultrasound attenuation and the backscatter, generating acoustic speckle on B-mode scans. The agar-based gelatin was produced using a solution very similar to the TMM one. However, the SiC and Al_2_O_3_ particles were omitted to avoid ultrasound back-scattering and speckle formation. The LV model was located at a depth of 5 cm (centre of the model), had a maximum chamber diameter of 4 cm, maximum wall thickness of approximately 1 cm and a chamber depth of 6 cm, typical values for a male human LV during the systole phase. Modest shape irregularities were deliberately introduced during construction of the LV structure to provide a more realistic model. When scanned along the *x-y* plane, the phantom simulates a snapshot in time of a parasternal short axis (PSAX) slice of a blood filled adult human LV.

### Data acquisition

The LV phantom was scanned using the Ultrasonix, Sonix-RP scanner (Ultrasonix Medical Corporation, Richmond, BC, Canada) along with an Ultrasonix PA2-4/20 (2–4 MHz) phased array cardiac probe. Due to restrictions imposed by the construction of the phantom, all data were acquired along the *x-y* plane simulating a PSAX slice of an adult human LV. Each dataset consisted of frames acquired from adjacent slices along the elevation plane with inter-slice angular displacement of 0.12° (two motor steps). One hundred and sixty-seven frames of adjacent slices formed the 3D acquisition sector. The angular 3D sector width was 19.92°, 9.96° from either side of the central acquisition plane. The pivot point was positioned on the distal end of the transducer, at the point of contact with the patient’s chest (depth = 0 mm). The probe frequency was set to 3.3 MHz, a typical frequency for acquiring adult human cardiac data. The scanning depth and beam focus were set at 9 and 5 cm, respectively, ensuring acquisition focusing within the LV structure. Other acquisition parameters such as sector width and gain (and Time Gain Compensation) were optimally set by the operator. Due to the nature of the Sonix RP (Ultrasonix) acquisition system, to cater for research in ultrasound imaging, data were acquired in fundamental mode with minimal pre-processing applied to the acquired B-mode images (besides the obvious conversion from the radio frequency signal to B-mode). The captured data were exported as DICOM image sequences of 600 × 800 pixels with no compression applied to them.

### Spatial compounding

The choice of spatial compounding method used has a direct effect on the processed images. Intensity averaging (mean or median) methods tend to generate a higher level of noise and speckle suppression. Techniques like maximum intensity compounding enhance anatomic feature delineation.^13^ More advanced approaches such as phase- and wavelet-based compounding provide a trade-off between noise suppression and feature delineation.^13^ Some techniques like maximum intensity, deconvolution, phase based and wavelet compounding have been shown to introduce a modest tissue/chamber contrast increase.^10,15^ In this paper, intensity averaging was utilised as a well-established, computationally efficient and effective spatial compounding method for noise suppression in ultrasound datasets. The intensity of each pixel within a compound frame I' was therefore set to the average intensity of the corresponding pixels from all the adjacent slices *I_i_*

(1) $$I'(x,y)=\frac{1}{N}\sum_{i=1}^{N}I_{i}(x,y)$$

where *N* was the number of adjacent slices utilised during compounding, and $I_{i}(x,y)$ represented the intensity of the pixel at location (x,y) within the *i*th slice in the volumetric dataset.

## Data analysis and results

**Figure 2.**
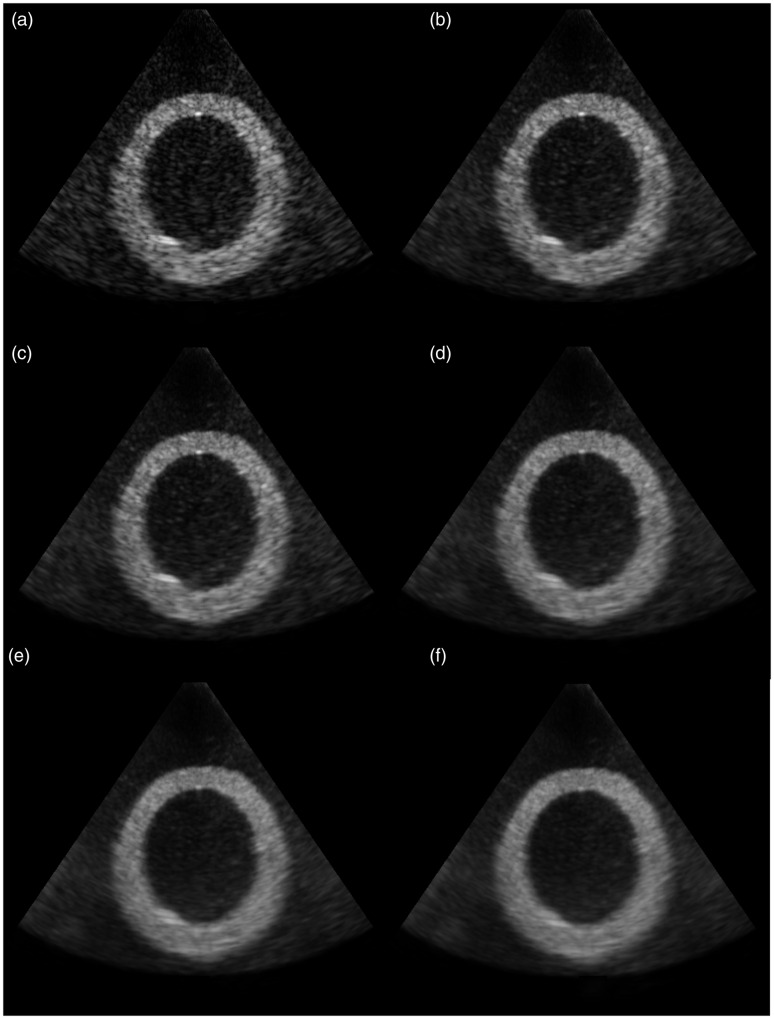
Original and compounded scans of LV phantom. (a) Original scan; (b)–(f) Compounded scans with 3D acquisition sector widths of (b) 2.4°; (c) 3.6°; (d) 7.2°; (e) 10.8° and (f) 14.4°.

Figure 2 displays a set of images acquired using the LV phantom. Figure 2(a) provides an unprocessed, B-mode scan of the *x-y* plane of the LV phantom. The remaining images in Figure 2(b) to (f) display compounded frames for increasing 3D acquisition sector widths ranging from 2.4° to 14.4°. In addition, a range of quantitative and qualitative results are presented assessing the effect of acquisition parameters such as inter-slice angular displacement and 3D sector angular range on speckle/noise suppression and tissue/chamber blurring.

### Effect on tissue SNR, chamber SNR, tissue/chamber contrast and signal difference to noise ratio

**Figure 3.**
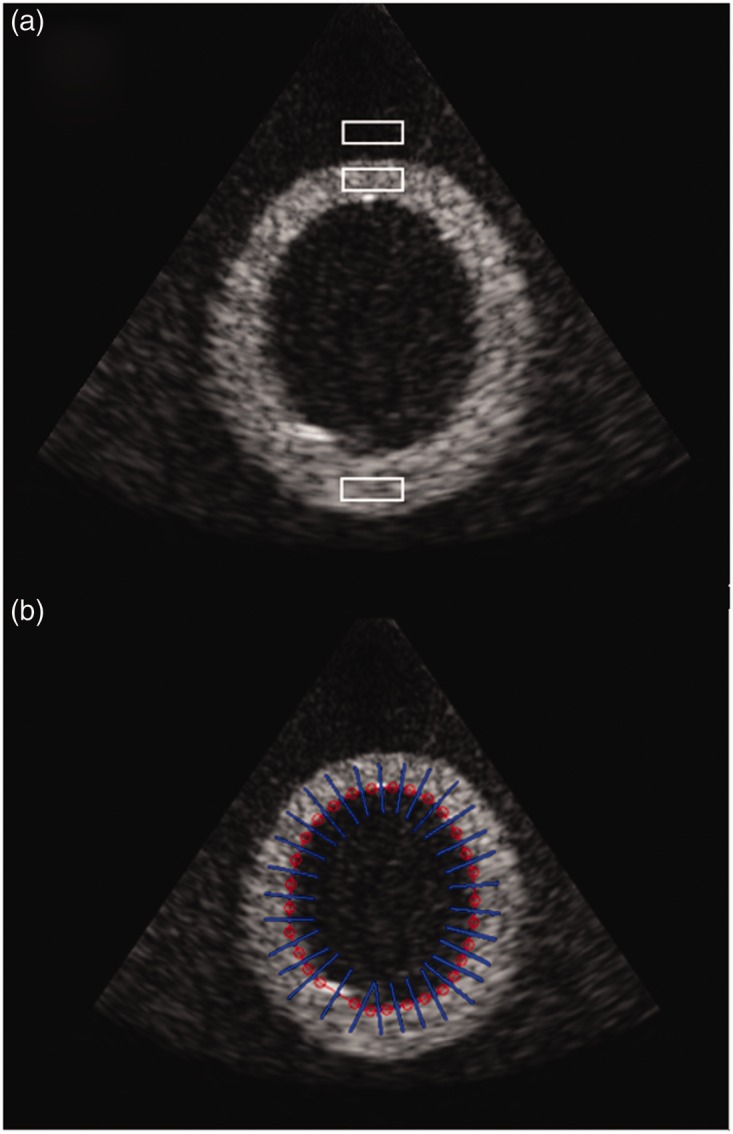
(a) Tissue and chamber ROIs. B-mode scan of LV phantom along with the rectangular ROIs used to derive the SNR, contrast and SNDR values. (b) LV outline and vertical perpendicular linear profiles. Manual outline of the LV chamber along with the linear segments (normal to the outline) used for the extraction of the intensity profiles necessary to examine the LV tissue boundary blurring.

Two rectangular regions of interest (ROI) of 11 × 35 pixels each, both corresponding to cardiac tissue, were defined (Figure 3(a)). The ROI on the top LV wall corresponds to the interventricular septum (IVS), while the ROI on the bottom LV wall corresponds to the LV posterior wall (LVPW). Similarly, a third ROI was defined above the LV structure representing the RV chamber in the parasternal short-axis view of the heart (Figure 3(a)). The pixel intensity values within each ROI were used to estimate the tissue and chamber SNRs on the original B-mode data as well as a series of compound images. SNR was estimated as the linear ratio

(2) $$\mathrm{SNR}=\bar{M}/\mathrm{SD}$$

where $\bar{M}$ and SD referred to the mean and standard deviation of the corresponding ROI intensity values.^3,36^ In a similar manner, the tissue/chamber contrast (C)^36,37^ and signal difference to noise ratio (SDNR), also referred to as detectability index,^36,38^ were derived as the following linear ratios

(3) $$C=\frac{|{\bar{M}}_{T}-{\bar{M}}_{C}|}{({\bar{M}}_{T}+{\bar{M}}_{C})/2}$$

(4) $$\mathrm{SDNR}=\frac{|{\bar{M}}_{T}-{\bar{M}}_{C}|}{{\mathrm{SD}}_{C}}$$

where ${\bar{M}}_{T}$ and ${\bar{M}}_{C}$ corresponded to the mean intensity level within the tissue and chamber ROIs, respectively, while ${\mathrm{SD}}_{C}$ corresponded to the chamber standard deviation. SDNR provides a very useful metric assessing how well a structure is displayed against a noisy background.

**Figure 4.**
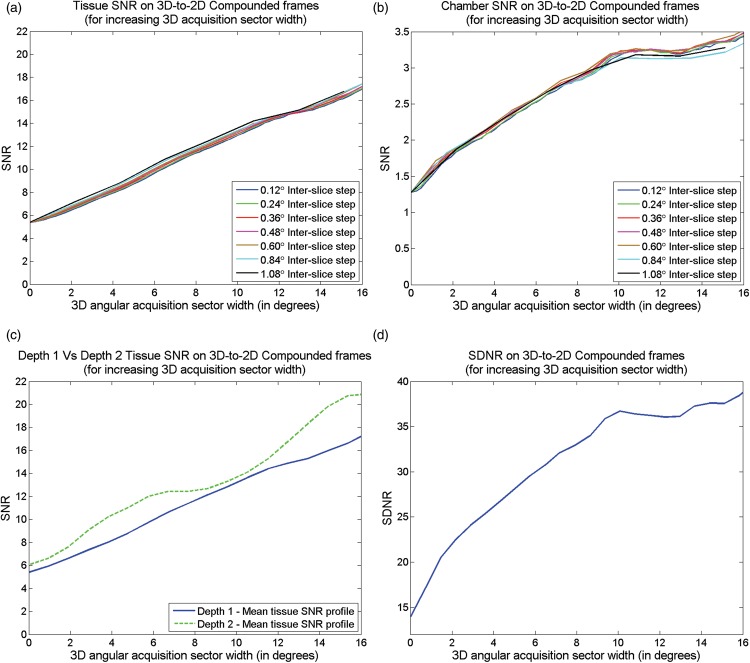
SNR and SDNR for increasing 3D acquisition sector width. The effect of ESC for increasing 3D acquisition sector width on (a) tissue SNR; (b) chamber SNR; (c) tissue SNR at IVS (Depth 1 – Top of LV) vs. SNR at LVPW (Depth 2 – Bottom of LV) and (d) tissue/chamber SDNR. SNR: signal to noise ratio; SDNR: signal difference to noise ratio.

Figure 4(a) and (b) display two plots with curves representing the tissue (top ROI corresponding to IVS) and chamber SNRs on the original (at the intercept with vertical axis) and a series of compounded images derived using ESC on data acquired with (i) an increasing 3D acquisition sector width and (ii) a range of inter-slice displacements. Since the inter-slice displacement during 3D acquisition is angular, the spatial inter-slice displacement increases with depth, possibly affecting the outcome of the compounding process. For that reason, Figure 4(c) provides a direct comparison between the SNR curves from both top and bottom tissue ROIs, utilising an intermediate inter-slice angular displacement of 0.36°. In a similar manner, Figure 4(d) displays a curve of the tissue/chamber detectability (SDNR) on the original, and a series of compounded images derived using increasing 3D acquisition sector width. Table 1 summarises the percentage change on each of the five quantitative measures between the original and compounded data for an increasing 3D acquisition sector width.

**Table 1.** Effect on image quality

Sector width	Tissue SNR (top)	Tissue SNR (bottom)	Chamber SNR	Contrast	SDNR
2.2°	24.90%	29.21%	46.63%	1.39%	60.86%
4.3°	54.11%	73.86%	76.78%	1.22%	91.74%
6.5°	91.31%	104.20%	107.11%	0.93%	120.18%
8.6°	123.41%	109.42%	130.60%	0.82%	143.40%
10.8°	155.19%	136.51%	153.10%	0.46%	160.77%
13.0°	177.79%	188.51%	149.80%	0.54%	158.73%
15.1°	203.37%	238.54%	162.84%	0.36%	169.10%

Mean overall effect of ESC on a range of quantitative measures for increasing 3D acquisition sector width expressed as a % increase compared to the values in the original single frame with no compounding. SNR: signal to noise ratio; SDNR: signal difference to noise ratio.

### Effect on tissue/chamber boundaries

**Figure 5.**
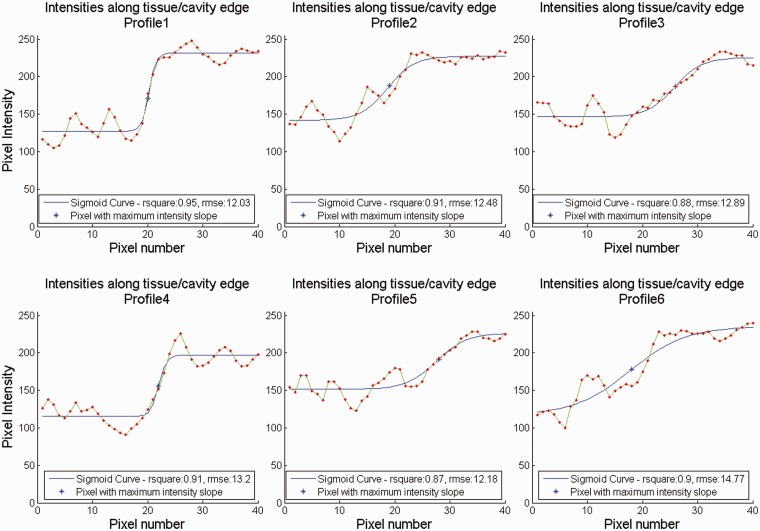
Tissue/chamber intensity profiles for original data. Intensity profiles along with the corresponding sigmoid curves at sample orientations of 0°, 60°, 120°, 180°, 240° and 300° (clockwise from top), respectively.

**Figure 6.**
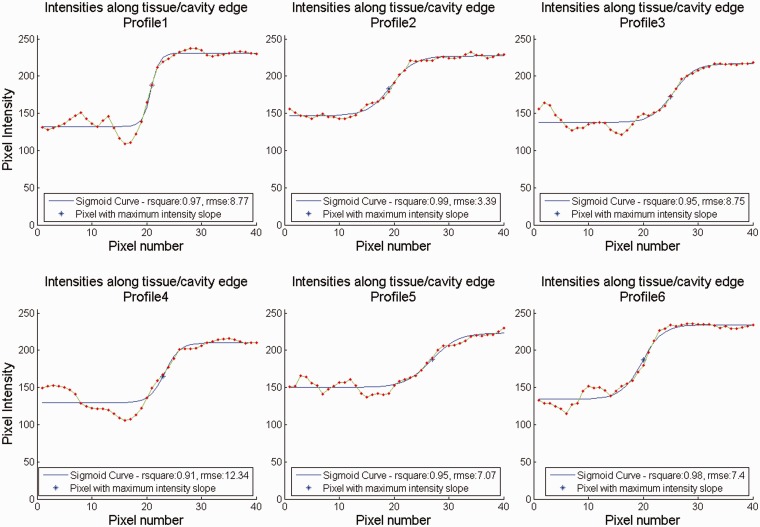
Tissue/chamber intensity profiles for compounded data (acquisition sector width of 7.2°). Intensity profiles along with the corresponding sigmoid curves at sample orientations of 0°, 60°, 120°, 180°, 240° and 300° (clockwise from top), respectively.

**Figure 7.**
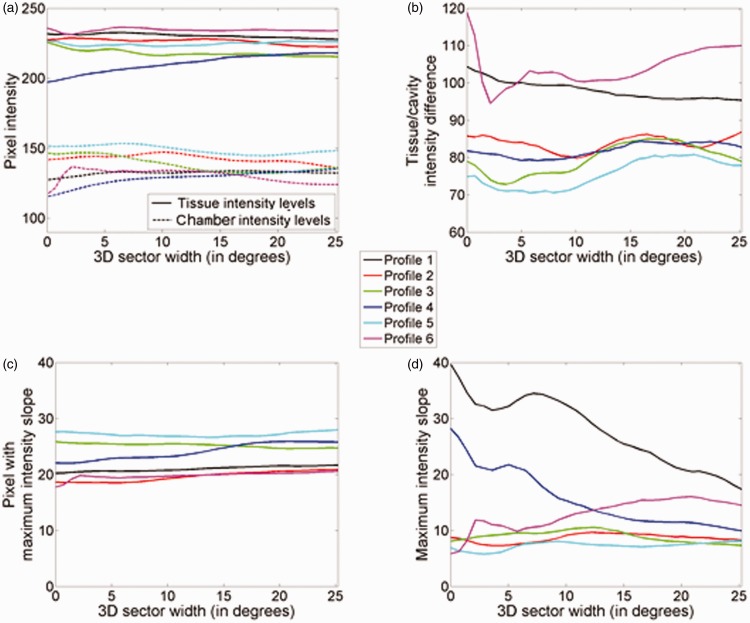
ESC and tissue/chamber blurring. Effect of ESC for increasing acquisition sector width on sigmoid curve parameters: (a) tissue vs. chamber intensity level; (b) tissue/chamber intensity difference; (c) position of pixel with maximum tissue/chamber intensity slope (d) maximum slope. Parameters for intensity profiles 1 to 6.

The LV chamber was manually outlined over the original B-mode scan, and intensity profiles were extracted at different depths and orientations along the tissue/chamber boundary. Each profile consisted of the intensity values of a 41 pixel linear segment along the normal of the outline (Figure 3(b)). Equivalent intensity profiles were extracted from a series of compounded images each derived using 3D data of increasing acquisition sector width and an inter-slice displacement of 0.36°. For the quantitative analysis of tissue/chamber boundary blurring, a sigmoid curve Y(t), modelled as a generalised logistic function^39,40^ was fitted through each intensity profile

(5) $$Y(t)=A+\frac{K-A}{1+e^{-B(t-M)}}$$

where t∈[1,41] is the position across the curve *Y*, *A* is the lower asymptote, *K* the upper asymptote, *M* the pixel at which maximum intensity growth occurs and *B* the relative growth rate at *M*. Figures 5 and 6 provide qualitative demonstrations of the effect of ESC on tissue/chamber boundary blurring. More precisely, six sample intensity profiles along with the corresponding sigmoid curves were extracted from the original (Figure 5) and a compounded image (Figure 6) (acquisition sector width of 7.2°) at orientations of 0°, 60°, 120°, 180°, 240° and 300° (clockwise from the top of the image). The extracted sigmoid curve parameters enable the quantitative assessment of the effect of ESC for increasing acquisition sector width on tissue/chamber boundary blurring. More precisely, *A* represents the mean chamber intensity, *K* represents the mean tissue intensity, *M* represents the point of tissue/chamber intersection and *B* represents the degree of tissue/chamber boundary blurring at *M*. The goodness of fit parameters, such as coefficient of determination (*R*^2^) and root mean square error (RMSE), also provide an indirect assessment of the amount of noise along the tissue/cavity boundary (for each sigmoid curve). The better the fit, the better defined the tissue/cavity boundary is. Figure 7 illustrates the effect of ESC on such parameters for increasing angular acquisition sector width. Each plot contains a curve for each of the six sample intensity profiles displayed in Figures 5 and 6.

## Discussion

### Effect on speckle and noise suppression

Through a visual inspection of the images displayed in Figure 2, it is apparent that ESC reduced the speckle in tissue as well as the noise in chamber areas of the LV phantom. The wider the acquisition sector, the larger is the speckle and noise suppression. This observation is verified by the quantitative results in Figure 4 and Table 1. More precisely, ESC had no noteworthy effect on tissue/chamber contrast. On the other hand, SNR of phantom tissue increased almost linearly with the 3D angular acquisition width utilised during ESC. Small variations in the SNR curves, such as in Figure 4(c), are attributed to inhomogeneities in the TMM as well as due to small errors in the mechanical motor-driven probe displacement. Furthermore, as illustrated in Figure 4(c), tissues at larger depths demonstrated larger SNR increases for any given angular acquisition width. This is due to the fact that for a given angular acquisition width, the corresponding spatial acquisition width increases with depth. Larger spatial acquisition width implies that the compounded slices will be less correlated, resulting in an increased SNR.

Figure 4(b) illustrates that similar to tissue, chamber SNR increased with the 3D angular acquisition width utilised during ESC. However, while for moderate acquisition widths (up to 10°), the SNR increase was substantial (153% for 10.8°) and almost linear; it levelled out as the 3D acquisition width increased (163% for 15.1°). A very similar behaviour was also observed in the tissue/chamber detectability index (SDNR) illustrated in Figure 4(d). This behaviour is attributed to the fact that unlike tissue, no new information on the cardiac chamber is introduced by each new adjacent slice. The noise suppression is solely based on random intensity variations present in cardiac ultrasound noise. Compounding of adjacent slices averages out such intensity variations, generating homogeneous cardiac chambers. Having achieved the intensity uniformity within cardiac chambers, compounding of additional slices has a minimal effect on the corresponding SNR and SDNR.

Finally, as demonstrated in Figure 4(a) and (b), the inter-slice angular distance has no noteworthy effect on speckle and noise suppression. This is due to the relation between speckle/noise suppression and data decorrelation. For small angular acquisition sectors, such as the sectors examined in this study, not much new information is introduced by further reducing the inter-slice acquisition distances. In addition, smaller inter-slice distance generates more data for a given acquisition sector width, increasing the technique’s computational requirements. On the other hand, for inter-slice distances larger than the acquisition beam width, speckle between the adjacent slices becomes fully decorrelated. Consequently, no additional information is gained during the compounding process by further increasing the acquisition sector width. Furthermore, larger inter-slice distances reduce the flexibility in the acquisition sector width choice in cases where the imaging window through the patient’s ribcage is restricted.

### Effect on tissue/chamber boundaries

Figure 2 illustrates that tissue/chamber boundary blurring was initially very moderate on data derived using narrow 3D acquisition sectors (Figure 2(a) to (c)), and it became more apparent as the 3D acquisition sector width increased (Figure 2(d) to (f)), especially at larger scanning depths (i.e., larger acquisition sector width). Figures 5 to 7 facilitate a better, quantitative understanding of the effect of ESC on tissue/chamber boundaries by analysing six individual intensity profiles across a representative range of depths and orientations. Due to the higher axial resolution of the acquisition system (when compared to lateral resolution), profiles 1 and 4 that lie along the direction of the ultrasound beam demonstrate a better defined tissue/chamber boundary than the rest of the boundaries (profiles 2, 3, 5 and 6). Furthermore, due to the closer proximity of profile 1 to the focal depth (when compared to profile 4), its original (unprocessed) tissue/chamber boundary is better defined than the corresponding boundary in profile 4. Figures 5 and 6 illustrate that by reducing noise and speckle across the tissue/chamber boundary, ESC increased the goodness of fit between the intensity profiles and their corresponding sigmoid curves, resulting in better defined boundaries (e.g., profiles 2, 3, 5 and 6). On the other hand, compounding could moderate the slope of the tissue/chamber transition, introducing tissue/chamber boundary blurring (e.g., profiles 1 and 4). Further examination of the individual parameters monitored in Figure 7 shows that ESC had a minor effect on the mean tissue and chamber intensities and therefore the associated tissue/chamber contrast (Figure 7(a) and (b)). Similarly, ESC had a very modest effect on the point of maximum slope (approximation of the tissue/chamber intersection) (Figure 7(c)). The variations in the corresponding curves were minimal with no potential clinical relevance. Moreover, examining the maximum intensity slope plots (Figure 7(d)) highlighted three distinctive behaviours. There are cases (e.g., profiles 2, 3 and 5) in which the compounding has no effect on the tissue/chamber boundary. There are cases in which the noise and speckle suppression (as seen in Figures 5 and 6) results in a better defined tissue/chamber boundary (e.g., profile 6). Finally, there are cases in which compounding results in blurring (decreasing slope) of the tissue/chamber boundary. The blurring is attributed to the deformations of the imaged structure’s cross section due to (i) the angular displacement of the imaging plane, combined with (ii) the shape variations/irregularities across the scanning sector width. An affine or non-linear spatial registration (prior to compounding) relating each point of each of the adjacent slices, *I_i_*, to the corresponding anatomical point in a reference image, *I_r_* (such as the central slice at *θ* = 0°), is expected to compensate for some of these deformations minimising tissue/chamber boundary blurring.

### Optimal acquisition set-up

The phantom provided a static and controlled environment (with optimal scanning conditions) to assess speckle/noise reduction and tissue/chamber boundary blurring. The findings should be therefore treated as a best case scenario since additional challenges (such as constant cardiac movement and a wide range of imaging artefacts) arise in real cardiac ultrasound scans. Furthermore, the scanning plane utilised throughout this study simulates a PSAX slice of a blood filled adult human LV. The orientation of the cardiac structure with respect to the scan plane ensures moderate shape variations along the elevational axis. Similar characteristics can be expected in the cardiac walls for most commonly used scan planes (both short- and long-axis). However, due to the shape of cardiac valves, some additional blurring can be expected when averaging across the elevational plane. Furthermore, due to the orientation of the aortic valve, in the PSAX basal view, the blurring can be severe. Similarly, the large curvature of the LV apical walls along the elevational plane may cause severe tissue/chamber blurring in the PSAX apical view.

For angles of up to 10°, sector width has a nearly linear relationship with the SNR and SDNR improvement in the acquired images. However, larger angular sectors require (i) larger acoustic windows through the rib cage and (ii) result in the compounding over larger spatial distances, increasing the risk of blurring the imaged structure (especially for the larger acquisition depths in long-axis scans). The nearly linear relation between the angular acquisition sector width and the improvement in both SNR and SDNR provides the clinician with the flexibility to choose an optimal sector width depending on the size and orientation of the imaged structure. Compounding data from a small 3D sector of 2.5° can still generate considerable data enhancement (potential SDNR increase of more than 60%). In scans through the long-axis apical view, with potential acquisition depths in excess of 15 cm and smaller structures such as valves imaged in the distal part of the scan, such small angular sectors may be preferable. On the other hand, in short-axis scans, which tend to be of lower acquisition depth and imaging mostly cardiac walls at the distal part of the scan, larger acquisition sector widths may be possible. Furthermore, since inter-slice displacement had no substantial effect in the resulting noise suppression, an intermediate option of 0.5° to 1.0° may reduce acquisition resources, while accommodating flexibility in the acquisition of small 3D segments when it is required.

As with any post-processing speckle suppression technique, under specific circumstances ESC can introduce some artefacts into the original data, degrading a structure of clinical interest. Flexibility on parameter selection (sector width), as well as the choice of the original and post-processed images during an examination can ensure that the clinician makes the most out of the available data.

## Conclusions

This paper extended the notion of ESC, a method for the acquisition and compounding of spatially adjacent, partially decorrelated slices, on cardiac ultrasound data. An anthropomorphic LV phantom was designed and used for the assessment of the effect of ESC on the imaged structures. ESC was found to suppress tissue speckle and chamber noise, substantially increasing the corresponding SNR as well as the tissue/chamber detectability index (SDNR). Speckle/noise reduction increases with the width of the 3D sector utilised during ESC. The noise and speckle reduction can help increase the detectability between cardiac tissue and chambers, enhancing boundaries that are masked by heavy levels of noise. On the other hand, ESC, by averaging adjacent and slightly deformed slices of a structure, can possibly introduce tissue/chamber boundary blurring. For narrow 3D sectors, the tissue boundary blurring appears to be negligible with no negative effect on the tissue/chamber edge enhancement introduced by noise reduction. However, for wider 3D sectors, the compounding of distant slices, where larger anatomic structure deformations occur, introduces tissue/chamber boundary blurring that counteracts the initial benefits introduced by the ESC of narrow acquisition sectors. Due to its effectiveness and simple nature (can be implemented for real-time applications with the use of matrix transducers), ESC demonstrates the potential to act as (i) a valuable adjunct to B-mode data echocardiographic examinations and (ii) a first step for post-processing techniques, such as image segmentation and registration, whose effectiveness is limited and sometimes restricted by low image quality (SNR and SDNR) in cardiac ultrasound data. Real-time implementation and further assessment (on volunteer and patient datasets) are required to evaluate the effect of ESC on the diagnostic value of cardiac ultrasound images.
